# Evaluation of the Beef Cattle Systems Model to Replicate a Beef Cow Genotype × Nutritional Environment Interaction

**DOI:** 10.3390/ani16030372

**Published:** 2026-01-24

**Authors:** Ivy Elkins, Phillip A. Lancaster, Robert L. Larson, Logan Thompson

**Affiliations:** 1 Beef Cattle Institute, Department of Clinical Sciences, Kansas State University, Manhattan, KS 66506, USA; ijschmid@vet.k-state.edu (I.E.); rlarson@vet.k-state.edu (R.L.L.); 2 Department of Animal Sciences and Industry, Kansas State University, Manhattan, KS 66506, USA; thom94@ksu.edu

**Keywords:** computer simulation, cow efficiency, maintenance energy requirements, reproduction

## Abstract

Cow efficiency is vitally important to beef sustainability, and computer simulation models may be useful tools to identify characteristics of the most efficient cow genotypes for a given production environment. The objective of this analysis was to determine whether the Beef Cattle Systems Model could replicate empirical research demonstrating a genotype–nutritional environment interaction for efficiency of feed conversion to calves weaned. Combinations of cow genotypes for lactation potential (8, 10, and 12 kg/d at peak milk) and growth potential (450, 505, and 650 kg mature weight) were simulated across four dry matter intake levels (58, 76, 93, and 111 g/kg BW^0.75^). At lower dry matter intakes, cows had lesser body condition scores and weight and longer postpartum intervals, but dry matter intake had minimal influence on pregnancy percentage or calf-weaning weight. These trends match empirical research except for pregnancy percentage, where decreasing dry matter intake had a dramatic effect on pregnancy percentage in high-milking, high-growth-potential genotypes. Efficiency of feed conversion was greatest at low dry matter intake for the model simulation with no evidence of a genotype–dry matter intake interaction, which is in contrast to empirical research demonstrating a genotype–dry matter intake interaction. In conclusion, standard nutrition equations do not replicate the genotype–nutritional environment interaction observed in empirical research studies.

## Simple Summary

The United States beef cow-calf sector consumes 70% of the feed resources and contributes approximately 70% of the carbon footprint to produce one kilogram of beef. Identifying beef cow genotypes that are more efficient at converting feed resources to weaned calves is vitally important to enhancing the sustainability of beef production. Previous research indicates that cow genotype interacts with the nutritional environment, indicating that large-scale research studies would be required to identify the most efficient genotype in the diverse nutritional environments across the U.S. The objective of this analysis was to determine whether a computer simulation model could replicate previous research demonstrating the genotype–nutritional environment interaction. Results indicate that the computer simulation model predicted a similar response of all genotypes across nutritional environments, indicating the absence of an interaction. In conclusion, current mathematical models estimate the performance of the average cow and are not substitutes for empirical research to identify the most efficient genotypes in diverse nutritional environments.

## 1. Introduction

Mismatches between cow genotype and nutritional environment can lead to suboptimal performance and increased costs [1]. When the genetic potential of the cow exceeds the available forage resources, performance suffers and production costs rise. Conversely, when the genetic potential is lower than the forage availability, resources are used inefficiently. Prioritizing the heaviest cows with the highest lactation potential has been a trend in the cow-calf industry; however, it may not necessarily enhance operational efficiency, as this approach can inadvertently exacerbate inefficiencies in the production cycle [2,3,4,5,6].

Breeding cows consume 70% of the total feed necessary for beef production, and 70% of this feed consumed by breeding cows is used for maintenance [4,7], such that 50% of the feed necessary for beef production is used to maintain the breeding cow. Additionally, cattle consuming forage-based diets have greater methane emissions, and the cow-calf sector of the beef production system accounts for approximately 70% of greenhouse gas emissions in North America [8,9]. Due to the expansive number of acres, the cow-calf sector also accounts for a disproportionately large share of other environmental impacts from beef production [8,10]. Thus, improvements in cow efficiency, the conversion of feed to kilograms of weaned calf, are critical to improving the sustainability of beef production.

Much of the focus on cow efficiency has been on mature cow size and lactation potential [4,6,11,12,13,14,15,16,17]. However, larger, heavier cows are not necessarily less efficient [18,19]. Rather, cow efficiency is dependent upon a genotype–nutritional environment interaction [20]. Cow efficiency involves partitioning of nutrients for maintenance including adaptation to the environment, body tissue gain/loss, reproduction, and lactation, which are polygenic biological processes. Previous omics research indicates that reproduction, lactation, body tissue growth, and maintenance are complex biological processes affected by many gene networks and metabolic pathways [21,22,23,24,25]. Additionally, cattle adapt to changes in their environment altering nutrient partitioning and efficiency [26,27,28,29].

However, conducting genotype–environment studies is difficult due to the large scale and long time frame necessary. Effective methods to identify the most efficient cow genotypes in a given forage availability and nutritional environment are lacking. Computer simulation models present a promising alternative to live animal trials for evaluating the genotype–environment interaction and identifying the genotype characteristics best matched to a given nutritional environment. Simulation models can be used to develop selection indices based on the contribution of important genotype characteristics [30], and deterministic computer models can be used to identify efficient cows within a herd [31].

The objective of this analysis was to evaluate the capability of a stochastic, dynamic computer simulation model to replicate the genotype–nutritional environment interaction reported by Jenkins and Ferrell [20]. The hypothesis was that a stochastic, herd-based simulation model would capture the dynamics of nutrition on reproduction efficiency, likely driving the genotype–nutritional environment interaction observed.

## 2. Materials and Methods

### 2.1. BCSM

The Beef Cattle Systems Model (BCSM) represents a cow-calf production herd in the Kansas Flint Hills based on U.S. trends in Angus EPDs from 1995 to 2019 [5]. The model is built to simulate an individual cow on a daily time step where the individual outcomes are influenced by the stochastic genotype selection (cow mature weight, lactation yield, postpartum interval), genetic and nutrition inputs, previous day’s outcomes, production status, and calendar date. The model generates the animal’s age, body weight, body condition score (BCS), lactation yield, reproductive condition, and morbidity and mortality rates. Equations from NASEM [32] are used to estimate nutrient requirements and intake, milk yield, and growth and body fat gain/loss. At the herd level, the model provides insights into nutrition, including forage, ration, and supplements; cow body condition and back fat; financial performance; profitability; calving and weaning metrics; reproductive efficiency; and data on culling and replacement heifers. A full compendium of the models’ capabilities can be found in Ahern [5].

The BCSM simulates a herd of 100 individual spring-calving heifers and cows for a maximum of twenty-four years. A production year is generated by the model for each breeding female; therefore, the length of each production year will vary based on the individual animal. A production year, as defined by the model, is the time from calving during the initial year to calving in the subsequent year or the date of culling from the herd. The breeding season start and end dates are identical between cows and heifers. Culling and replacement decisions are made based on the production calendar date and the daily feedback provided by the animal characteristics output on an individual and herd basis. Animals that stay in the herd from year to year change age status. Animals enter the production cycle as a nursing calf and progress through post-weaning replacement heifer, bred heifers, two-year-old cows, three-year-old cows, and mature cows (greater than four years old) and cycle through conditions of pregnant and non-pregnant, estrus and anestrus, and lactating and non-lactating. Culling decisions are made after evaluation of pregnancy, 60 days post-breeding. Any female whose calf dies before the start of the breeding season is removed from the herd immediately. Nonpregnant cows and those that exceed 13 years of age at the time of pregnancy diagnosis are culled and sold at calf weaning. All calves are weaned and sold on the date that the oldest calf is 220 days old. Additionally, if a female aborts her pregnancy following the cull date, she is removed from the herd on that date. The model establishes minimum culling thresholds for each age group of cows. If the percentage of open females does not meet the minimum culling threshold for each individual age group, cows will be culled until the minimum threshold is achieved. Replacement heifers are raised internally and selected in order of age, with preference to older heifers. If there is additional demand that exceeds the raised heifer crop, heifers are purchased to match traits of the existing herd.

### 2.2. Parameterization for This Study

#### 2.2.1. Genetics

Cow genotypes with respect to mature shrunk body weight (MSBW) and peak lactation were parameterized to simulate the cow genetics reported by Jenkins and Ferrell [20,33]. The mean mature shrunk body weights used were 450, 550, and 650 kg, closely matching the range of cow weights reported for the different breeds used by Jenkins and Ferrell [20]. Peak lactation yields were set at 8.0, 10.0, and 12.0 kg/d to match the median peak lactation and encompass the range of peak lactations reported for the different breeds by Jenkins and Ferrell [33]. Each MSBW genotype was simulated with each peak lactation genotype, resulting in 9 cow genotypes. The MSBW of each individual cow was drawn from a distribution having 1 of the 3 mean MSBW listed above and a standard deviation of 25.4 kg, and similarly the peak lactation potential of each individual cow was drawn from a distribution having 1 of the 3 mean peak lactation yields listed above and a standard deviation of 1.45 kg [5]. Calf birth weight and weaning weight estimated breeding values were adjusted, assuming sire mature cow weight was equal to the mean mature cow shrunk body weight above and did not increase over time (1995–2019) as in the original BCSM because the mean mature cow shrunk body weight did not increase over time.

#### 2.2.2. Management

**Figure 1:**
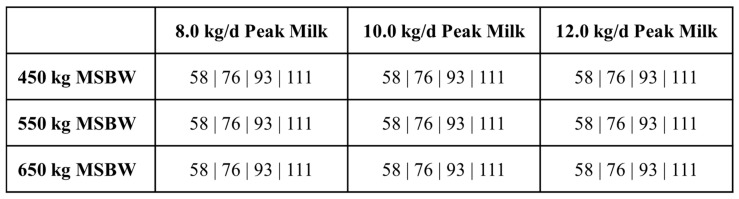
Genotype–nutritional environment scenarios simulated by the Beef Cattle Systems Model. Values represent feed intake as g/kg^0.75^ MSBW. MSBW = cow mature shrunk body weight.

To match management used by Jenkins and Ferrell [20], the start of the breeding season was set at June 15 and lasted for 90 days. Grazed forage was removed from the BCSM, and cows were modeled as if fed a totally mixed ration in a drylot. Three-year-old, four-year-old, and mature cows were assigned a diet with a metabolizable energy content of 2.25 Mcal/kg DM with a formulation as reported by Jenkins and Ferrell [6]. As Jenkins and Ferrell only reported diets for 3-year-old and older cows, a formulated diet was then developed for nursing calves, post-weaning replacement heifers, bred heifers, and two-year-old cows (Table S1). Each 3+-yr-old cow genotype was simulated to have a DMI of 58, 76, 93, and 111 g/kg^0.75^ MSBW with an additional 18 g/kg^0.75^ during lactation (Figure 1). For DMI calculation, MSBW for three-year-old cows was adjusted to 96% of their mature body weight, whereas MSBW for four-year-olds was adjusted to 98% based on target weight at calving for three- and four-year-old cows [9]. As reported by Jenkins and Ferrell [20], cows were fed 7 kg DM per day of corn silage approximately 14 days before calving season was to start instead of the experimental diet and designated amounts. Each cow was again fed the experimental diet and designated amount starting 10 days after calving.

Weight gain/loss was estimated from the positive or negative energy balance (net energy required–net energy intake) on each day using increasing energy content of gain/loss with increasing empty body fat percentage (Table S2). The change in empty body fat percentage per kilogram of weight change was used to determine the empty body fat percentage on each day, which was used to determine the body condition score on each day.

Lancaster and Larson [34] demonstrated that the original BCSM underestimated daily calf forage intake and growth within the pre-weaned calf nutrition subroutine. Building on this, Baldin [35] developed more precise prediction equations that better reflect empirical data. As a result, the BCSM was revised to integrate the equations of Baldin [35]. Additionally, equations for predicting gross energy production during lactation were integrated into the BCSM [35]. Jenkins and Ferrell [20] reported that calves were provided 2 or 3 kg per calf per week of creep feed starting when the oldest calf within a pen was 120 days of age. The calf forage intake equation predicts considerably greater creep feed intake than 2 or 3 kg per week, and we assume that calves did not consume all of the feed at the start. Given that the BCSM simulates each individual animal, we parameterized the model to simulate creep feed consumption starting at 75 days of age and adjusted the predicted intake using a multiplication factor of 0.2, which was found by iteration to give creep feed intakes close to 3 kg per week on average, and also scaled creep feed intake as milk production declined and calf body weight increased throughout the nursing period.

Dry matter intake of post-weaning replacement and post-breeding bred heifers was set equal to dry matter required to achieve target body weights of 60% and 80% of MSBW at breeding and first calving, respectively. Dry matter intake of 2-year-old cows was predicted using equations from NASEM [32].

### 2.3. Data Analysis

Model outputs were computed to provide similar data as presented by Jenkins and Ferrell [20] for comparison based on cows 3 years old or older. Individual outputs included cow body weight at calving and the subsequent calving and cow body condition at calving, peak lactation, weaning, and the subsequent calving. A mean body weight or median body condition score was computed for the herd. Total DMI for the production year was outputted from the model for individual cows and summed for the herd. The postpartum interval for individual cows was outputted from the model, and the mean postpartum interval was computed for the herd. The number of heifers kept, 3+-year-old cows bred and calved, and calves weaned for the herd were outputted from the model. Reproductive measurements were calculated and included pregnancy, calving, and weaning percentages, and the percent of cows cycling in the first 21 days of the breeding season. Variables for the herd were averaged across production years to obtain a single value for each cow genotype and DMI level scenario.

Polynomial regression analysis was used to evaluate relationships of DMI level with reproduction (postpartum interval, percent cycling in the first 21 d of breeding season, pregnancy percentage, calving percentage, weaning percentage), production (cow body condition score, cow body weight, pre-weaning average daily gain, weaning weight, weaning weight per cow exposed), and efficiency (grams of calf weaned per kilogram of DMI per cow exposed) of 3+-year-old cows. Regression analysis was performed using *lm* function in the base package of R (version 4.2.1; R Core Team) to estimate coefficients; statistical tests are not presented as these are computer simulation data. Given the nature of simulated data, traditional statistical analysis is not relevant. Interpretation of regression slopes was based on expert knowledge of biologically meaningful changes.

## 3. Results

The purpose of this analysis was to evaluate whether the BCSM could accurately simulate the cow genotype–nutritional environment interactions reported by Jenkins and Ferrell [20]. Results of Jenkins and Ferrell [20] are adapted and presented alongside outputs from the BCSM simulations for ease of comparison. Jenkins and Ferrell [20] used cows with mature weights ranging from 474 to 675 kg and peak milk yields ranging from 8.5 to 11.9 kg/d [33], which is similar to the mature cow weights ranging from 450 to 650 kg and peak milk yields ranging from 8.0 to 12.0 kg/d used in the BCSM simulations.

**Figure 2:**
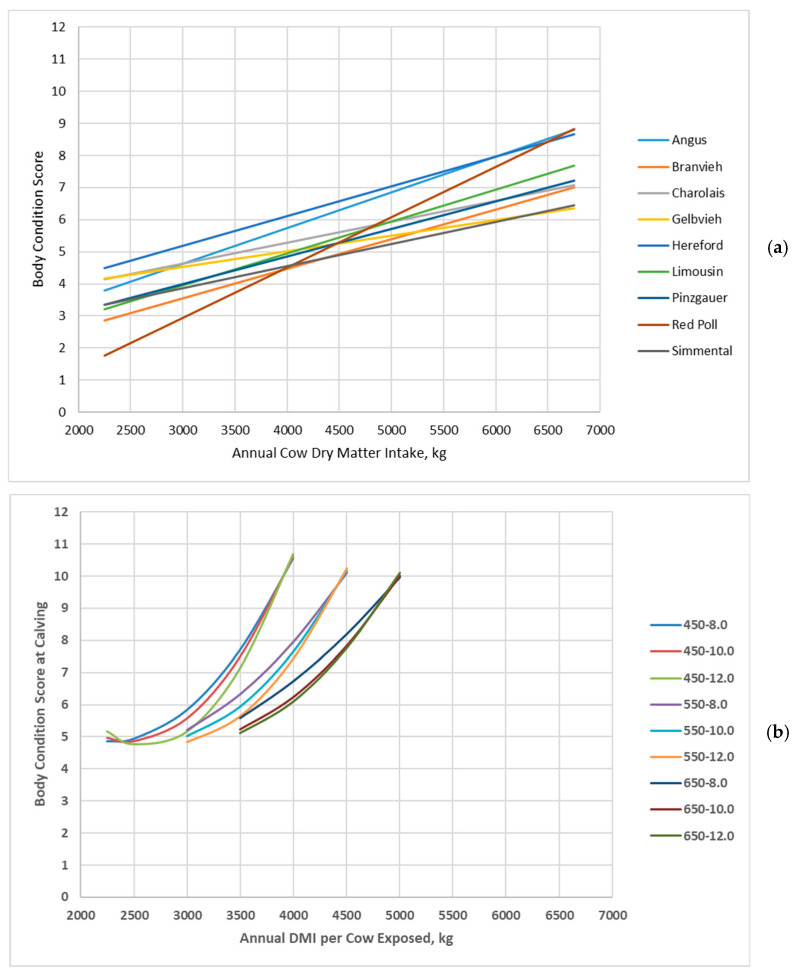
Body condition score of cow genotypes in relation with annual cow dry matter intake: (**a**) Data adapted from Jenkins and Ferrell [20]; (**b**) Data simulated by the Beef Cattle Systems Model. Panel (**a**): Angus, Braunvieh, Charolais, Gelbvieh, Hereford, Limousin, Pinzgauer, Red Poll, and Simmental refer to breeds of cows used by Jenkins and Ferrell [20]. Panel (**b**): 450-8.0, 450-10.0, 450-12.0, 550-8.0, 550-10.0, 550-12.0, 650-8.0, 650-10.0, and 650-12.0 represent the combinations of mature shrunk body weight (MSBW) and peak milk yield (e.g., 450 MSBW-8.0 peak milk) used in Beef Cattle Systems Model simulations.

Jenkins and Ferrell [20] reported that body condition score linearly increased with increasing annual dry matter intake (Figure 2a), but there were differences in the slope among cow genotypes. In the BCSM outputs, body condition score at calving increased at an increasing rate with increasing annual dry matter intake for almost all cow genotypes (Figure 2b). Cows achieved greater BCS at high annual dry matter intake in the BCSM than for Jenkins and Ferrell [20]. Also, cows did not lose as much body condition at lesser annual dry matter intakes in the BCSM, with cows remaining at a BCS of 4 or greater compared to Jenkins and Ferrell [20], who reported some cow genotypes decreasing to a BCS of 2 or 3.

**Figure 3:**
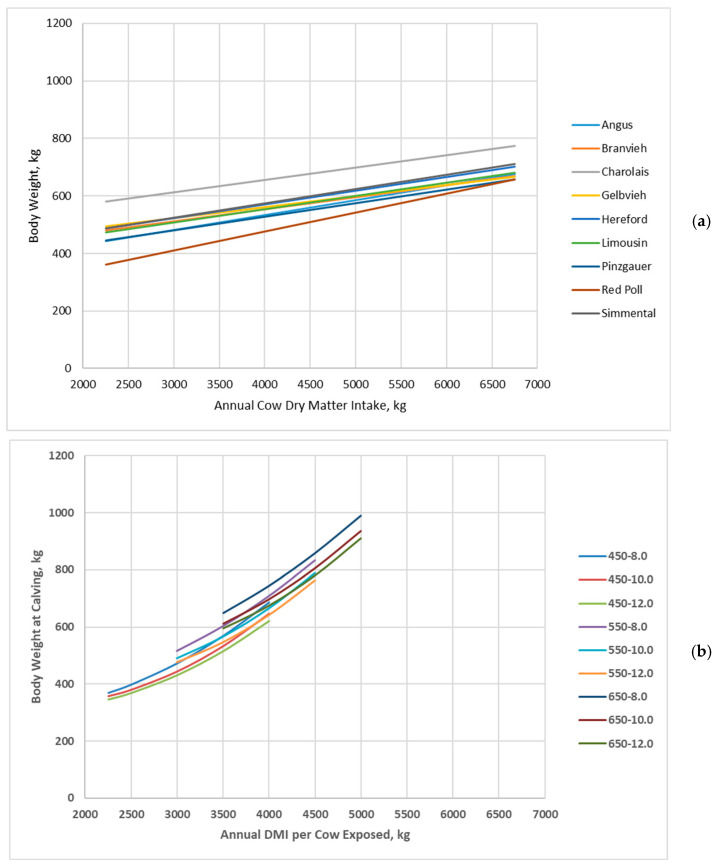
Body weight of cow genotypes in relation with annual cow dry matter intake: (**a**) Data adapted from Jenkins and Ferrell [20]; (**b**) Data simulated by the Beef Cattle Systems Model. Panel (**a**): Angus, Braunvieh, Charolais, Gelbvieh, Hereford, Limousin, Pinzgauer, Red Poll, and Simmental refer to breeds of cows used by Jenkins and Ferrell [20]. Panel (**b**): 450-8.0, 450-10.0, 450-12.0, 550-8.0, 550-10.0, 550-12.0, 650-8.0, 650-10.0, and 650-12.0 represent the combinations of mature shrunk body weight (MSBW) and peak milk yield (e.g., 450 MSBW-8.0 peak milk) used in Beef Cattle Systems Model simulations.

Cow body weight linearly increased with increasing annual dry matter intake, and cow genotype interacted with annual dry matter intake for Jenkins and Ferrell [20] (Figure 3a). Likewise, cow body weight at calving increased with increasing annual dry matter intake for all cow genotypes in the BCSM; however, all cow genotypes increased at similar rates with increasing annual dry matter intake (Figure 3b). Also, cow body weight more rapidly increased with increasing annual dry matter intake and to a greater extent at greater annual dry matter intakes in the BCSM compared to Jenkins and Ferrell [20].

**Figure 4:**
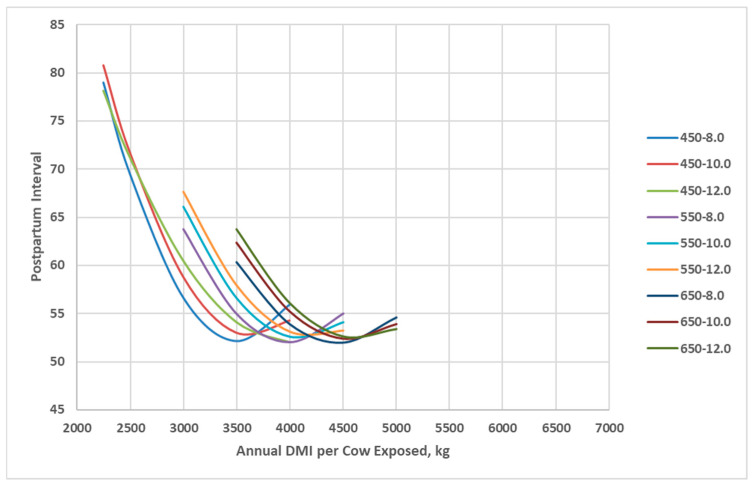
Postpartum interval of cow genotypes in relation with annual cow dry matter intake as simulated by the Beef Cattle Systems Model. 450-8.0, 450-10.0, 450-12.0, 550-8.0, 550-10.0, 550-12.0, 650-8.0, 650-10.0, and 650-12.0 represent the combinations of mature shrunk body weight (MSBW) and peak milk yield (e.g., 450 MSBW-8.0 peak milk) used in Beef Cattle Systems Model simulations.

In the BCSM, postpartum interval is randomly selected from a triangular distribution (min, mode, max) based on BCS at calving with a minimum mode value of 50 days for BCS 6 and greater. The postpartum interval increased as annual dry matter intake decreased for all cow genotypes, with 450 kg cows having a peak milk yield of 10 kg/d achieving the greatest postpartum interval at low annual dry matter intake (Figure 4). Cows with greater lactation potential required greater annual dry matter intake to achieve similar postpartum intervals.

**Figure 5:**
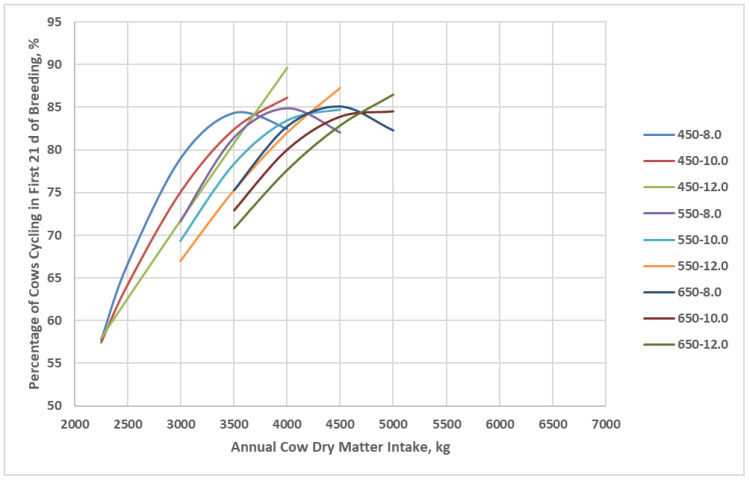
Percent of cows cycling in the first 21 d period of the breeding season of cow genotypes in relation to annual cow dry matter intake as simulated by the Beef Cattle Systems Model. 450-8.0, 450-10.0, 450-12.0, 550-8.0, 550-10.0, 550-12.0, 650-8.0, 650-10.0, and 650-12.0 represent the combinations of mature shrunk body weight (MSBW) and peak milk yield (e.g., 450 MSBW-8.0 peak milk) used in Beef Cattle Systems Model simulations.

As a result of differences in postpartum interval, the percentage of cows cycling in the first 21 d of the breeding season increased with increasing annual dry matter intake for the BCSM (Figure 5). At high annual dry matter intake, the percentage of cows cycling in the first 21 d of the breeding season plateaued near 85% for all cow genotypes with less than 12 kg/d peak milk yield. The percentage of cows cycling in the first 21 d of the breeding season had less curvature for cow genotypes with a peak milk yield of 12 kg/d, indicating that at high peak milk yield, a larger percentage of the cows were not cycling at high dry matter intake, resulting in minimal quadratic response.

There was not a cow genotype–dry matter intake level interaction for pregnancy, calving, and weaning percentage; thus, average BCSM outputs are presented for each cow genotype in Table 1, and average BCSM outputs are presented for dry matter intake levels in Table 2. Cow genotype had no influence on pregnancy, calving, or weaning percentage; however, at 58 g/kg^0.75^ dry matter intake, pregnancy, calving, and weaning percentage were reduced slightly. This is in contrast to results of Jenkins and Ferrell [20], who reported that calving rate dramatically decreased at lower levels of dry matter intake for most, but not all, cow genotypes. Even though the postpartum interval increased and the percentage of cows cycling in the first 21 d of the breeding season decreased at lesser annual dry matter intakes in the BCSM, simulating the 90 d breeding season used by Jenkins and Ferrell [20] allowed time for most cows to resume estrus, resulting in pregnancy rates near 95%.

Birth weight increased with increasing cow MSBW (Table 3). Creep dry matter intake by nursing calves was similar among dry matter intake levels for all cow genotypes, but creep dry matter intake increased with increasing cow MSBW and increasing peak milk yield. Weaning age was similar among cow genotypes due to being programmed into the BCSM as the point the first-born calf reaches 220 days of age. Similar to Jenkins and Ferrell [20], pre-weaning ADG and weaning weight were not affected by cow dry matter intake level but differed among cow genotypes. In both the BCSM simulation and the study by Jenkins and Ferrell [20], calves from cow genotypes with greater MSBW and peak milk yield had increased pre-weaning ADG and weaning weight. In contrast to Jenkins and Ferrell [20], weaning weight per cow exposed from the BCSM was not affected by the cow genotype–dry matter intake level interaction, which is likely due to the lack of change in pregnancy and calving percentages with dry matter intake level. Weaning weight per cow exposed from the BCSM increased with increasing cow MSBW and peak milk yield.

**Figure 6:**
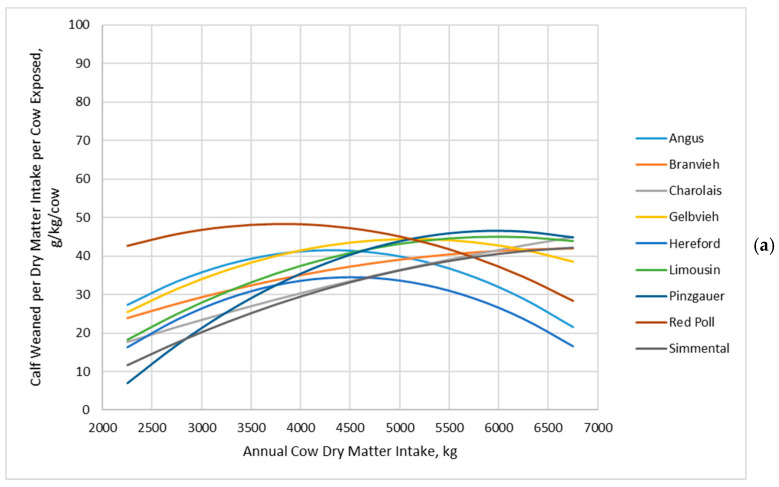

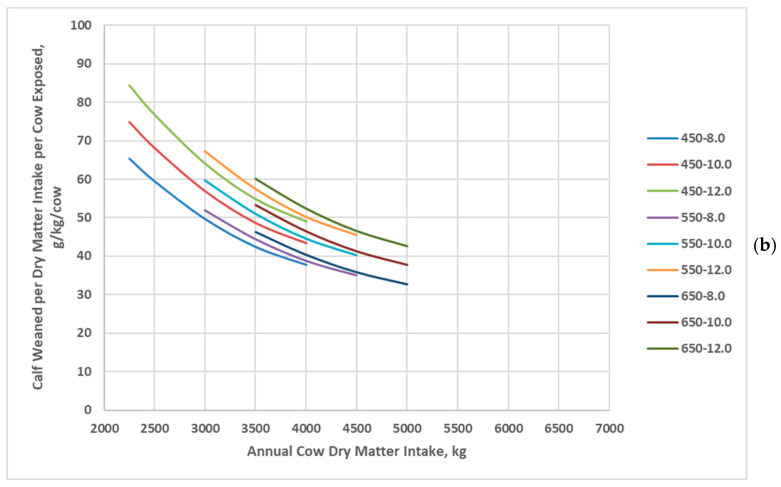
Grams of calf weaned per kilogram of dry matter intake per cow exposed of cow genotypes in relation with annual cow dry matter intake: (**a**) Data adapted from Jenkins and Ferrell [20]; (**b**) Data simulated by the Beef Cattle Systems Model. Panel (**a**): Angus, Braunvieh, Charolais, Gelbvieh, Hereford, Limousin, Pinzgauer, Red Poll, and Simmental refer to breeds of cows used by Jenkins and Ferrell [20]. Panel (**b**): 450-8.0, 450-10.0, 450-12.0, 550-8.0, 550-10.0, 550-12.0, 650-8.0, 650-10.0, and 650-12.0 represent the combinations of mature shrunk body weight (MSBW) and peak milk yield (e.g., 450 MSBW-8.0 peak milk) used in Beef Cattle Systems Model simulations.

Jenkins and Ferrell [20] reported that cow genotype interacted with annual dry matter intake to affect cow efficiency (grams of calf weaned per kilogram of dry matter intake per cow exposed), with lighter MSBW and lesser milk yields being more efficient at low annual dry matter intake but less efficient at high annual dry matter intake (Figure 6a). In contrast, cow efficiency (grams of calf weaned per kilogram of dry matter intake per cow exposed) decreased at a decreasing rate with increasing annual dry matter intake for all cow genotypes in the BCSM simulation (Figure 6b).

## 4. Discussion

Simulating cows of various genetic potentials for growth/mature size and lactation fed various levels of dry matter intake using the BCSM resulted in different results for grams of calf weaned per kilogram of dry matter intake per cow exposed (i.e., cow efficiency) than the empirical trial by Jenkins and Ferrell [20]. There are several potential reasons for the discrepancy between studies related to the weaning weight of calves and the reproductive efficiency of cows exposed, which are outlined in the following discussion.

In both the BCSM simulation and the study by Jenkins and Ferrell [20], weaning weight varied with cow mature weight and milk yield but was not affected by annual cow dry matter intake. Weaning weight per calf was highly driven by milk yield in the BCSM, but weaning weight is only moderately correlated with milk yield in previous studies [36,37,38,39,40,41,42]. In some instances, calves receiving low milk production from the dam will compensate with increased forage intake [43], which is somewhat accounted for in the creep feed intake equation used in the BCSM [5,35,44,45]. However, in the BCSM, the milk yield equation is not connected to the energy intake of the cow and computes a constant lactation curve across cow annual dry matter intake levels; thus, the predicted feed intake of the calf is strictly a function of calf body weight at a given lactation potential regardless of cow dry matter intake.

Biologically, milk production may be dynamic in relation to energy intake and gluconeogenesis necessary for lactose synthesis [46,47,48,49,50]. Milk yield is modeled in the BCSM as cows achieve genetic potential for lactation regardless of nutrient intake, resulting in negative energy balance when the net energy required for lactation is greater than net energy intake above maintenance. However, there is evidence that beef cows will decrease milk yield when energy intake is reduced [33,51,52,53,54]. Thus, the cow efficiency estimated by the BCSM at low dry matter intake levels may be overestimated due to a lack of reduction in milk yield resulting in decreased calf growth.

The largest discrepancy between the current simulation with the BCSM and the study by Jenkins and Ferrell [20] is the calving percentage of cows at low dry matter intake levels. In the study by Jenkins and Ferrell [20], cows with greater growth potential had a large decrease in calving percentage at low dry matter intake levels, which was not observed in the BCSM simulation. Calving percentage is a consequence of pregnancy percentage, which is a consequence of postpartum interval that is a consequence of body condition score at calving. Body condition score is a consequence of energy intake relative to maintenance and lactation energy requirements.

In the BCSM simulation, cows gained more weight and body condition score at high levels of dry matter intake and lost less weight and body condition at low levels of dry matter intake than in the study by Jenkins and Ferrell [20], which is likely a function of maintenance energy requirements. Maintenance energy requirements are computed on a net energy basis (Equations (S1)–(S3)) based on NASEM [32]. Basal net energy for maintenance is based on metabolic body weight; however, net energy required for maintenance is affected by body composition. Protein content of the carcass and viscera are strongly positively related to fasting heat production [55,56,57,58,59,60] and maintenance energy requirements [61]. Breed types with greater lean mass and chemical protein content have greater fasting heat production [58,62]. In the BCSM, cows of different mature weights are essentially assumed to be of similar body composition, whereas, in the study by Jenkins and Ferrell [20], breeds differed in body composition and the change in composition with increasing dry matter intake levels [63].

Fasting heat production at a point in time is affected by the previous plane of nutrition, which impacts the size of visceral organs [55,57,59,64,65,66,67]. Metabolizable energy intake affects the size of visceral organs, particularly the liver [65,66,68,69,70,71], which accounts for approximately 40 to 50% of whole-body energy expenditure [72,73,74]. However, visceral organ mass is a relatively small percentage (~10%) of empty body weight [72], indicating that fasting heat production per unit of metabolic weight increases with increasing metabolizable energy intake. In the BCSM, maintenance energy requirements are computed based on a constant net energy required for a maintenance factor of 0.077 Mcal/kg^0.75^ (Equation (S3)), and any effect of dry matter intake level on visceral organ mass that would be inherent in the results of Jenkins and Ferrell [20] is not incorporated in the BCSM due to a lack of prediction equations.

In the BCSM simulation, daily dry matter intake was computed based on mature cow size (450, 550, or 650 kg) and was constant for a given cow throughout her lifetime, which is our interpretation of the experimental procedure used by Jenkins and Ferrell [20], although it is not explicitly stated. Net energy for maintenance requirements was computed based on the current body weight. Thus, as cows lost weight at low dry matter intake levels, the dry matter intake remained constant and the maintenance energy requirement decreased, slowing the loss in body weight and condition, and inversely, as cows gained weight at high dry matter intake levels, the dry matter intake remained constant and the maintenance energy requirement increased, slowing the gain in body weight and condition, which somewhat simulated the potential decrease and increase in maintenance energy requirements relative to feed intake that would be expected with changing visceral organ mass. However, the constant dry matter intake along with the changing maintenance energy requirement did not fully replicate changes in body weight and body condition observed with various dry matter intake levels observed by Jenkins and Ferrell [20].

The net energy required for maintenance is greater for dairy breeds than beef breeds of cattle [32,46,75], and beef breeds with greater lactation potential have increased maintenance energy requirements even when not lactating [75,76,77,78,79]. In the BCSM, the net energy required for maintenance for cow genotypes with peak milk yield less than 7 kg/d was decreased 12%, assuming that the baseline net energy value (0.077 Mcal/kg^0.75^) was established with cows having peak milk yield greater than 7 kg/d (Equation (S3)). The 12% adjustment was based on the work of Montaño-Bermudez et al. [76], but further investigation may be necessary to refine this estimate. Other studies have demonstrated that breed types with greater lactation potential have 20 to 30% greater energy requirements to maintain body weight [75,77], and the adjustment may not be a simple distinction at a particular peak milk yield but rather a continuum [80].

Efficiency of metabolizable energy use for conceptus growth has been estimated to be approximately 13% [32,81], but the low efficiency is partly due to increased energy expenditure by the maternal tissues [82,83], particularly the liver [84], to maintain the greater reproductive tissue mass, accounting for approximately 50% of the increased heat production during gestation [85]. In the BCSM, the net energy required for gestation is adjusted for low efficiency and converted to a net energy for maintenance basis (Equation (S1)), which partially accounts for the increased maintenance associated with gestation. However, adjustment is based on a calculated partial efficiency of metabolizable energy use for maintenance, which assumes that efficiency of metabolizable energy use for maintenance is purely a function of the diet. There are inconsistent data and theories on whether animal factors affect the partial efficiency of metabolizable energy use [86,87,88,89]; and thus, there is little information to make mathematical adjustments in the BCSM.

In the study by Jenkins and Ferrell [20], Angus, Hereford, and Red Poll cows were the most efficient (grams of calf weaned per kg of dry matter intake per cow exposed) at low dry matter intake, which is likely due to the fact that these breeds maintained high calving percentages, even at low dry matter intake. Evaluating these three breeds as a group, Nugent et al. [90] reported shorter postpartum intervals at low dry matter intakes than the other breeds. At low dry matter intake, Angus and Hereford cows had greater empty body fat percentage and lower empty body protein percentage than other breeds [62], which may have lowered maintenance energy requirements [91,92] and/or maintained greater fertility [93,94,95,96,97,98] due to the increased body fat. Hereford cows had lower heat production at low dry matter intake than Simmental cows [62].

Interestingly, Red Poll cows had very low body condition scores and empty body fat percentages at low dry matter intake; however, they maintained high calving percentages. The mechanism by which Red Poll cows maintained high calving percentages at low dry matter intake is not readily apparent. Red Poll cows had greater visceral organ mass (g/kg empty body weight) at low dry matter intake than other breeds [63], indicating that maintenance energy requirements were likely not reduced at low dry matter intake. In fact, non-pregnant, non-lactating Red Poll cows maintained lesser kg of body weight per kg of feed at each of the feed intake levels [99], indicating greater maintenance energy requirements. Red Poll cows had moderate milk yield among the breeds evaluated [33] but have been reported to have lesser butterfat concentration [100]. Potentially, Red Poll cows could have reduced milk yield or milk fat at low dry matter intake, thus allowing more energy for reproduction in early lactation, although there was no evidence of breed by dry matter intake interaction on milk yield [33].

Conversely, Angus, Hereford, and Red Poll cows were less efficient (grams of calf weaned per kg of dry matter intake per cow exposed) at high dry matter intake than other breeds. The greater efficiency of Charolais, Simmental, Braunvieh, Gelbvieh, Pinzgauer, and Limousin cows could be due to increased mature weight and growth potential of their calves; however, weaning weight per calf weaned was not affected by cow annual dry matter intake [20]. The difference is likely due to the increased calving percentage, and thus the larger mature weight and growth potential were evident. Nugent et al. [90] reported that biological types with high growth potential (Charolais, Limousin) had the greatest positive response in postpartum interval to increased dry matter intake. There were little differences in visceral organ mass at high dry matter intake among breeds, but Braunvieh, Charolais, Simmental, Gelbvieh, and Limousin had lesser empty body fat percentage than Angus, Hereford, and Red Poll cows [63]. Additionally, Jenkins and Ferrell [98] reported greater body weight maintained per kg of feed intake at high dry matter intake for Limousin and Pinzgauer cows, but not Charolais, Gelbvieh, Braunvieh, or Simmental cows compared with Angus, Hereford, and Red Poll cows. However, Simmental cows had lesser heat production at high dry matter intake than Hereford cows [62], indicating greater energy available for reproduction.

The rate of change in heat production with increasing dry matter intake was greater for Hereford cows than Simmental cows [62], indicating differences in heat increment. There was not a difference in change in visceral organ mass with increasing dry matter intake between Hereford and Simmental cows [63]. However, Simmental cows gained more empty body protein but similar empty body fat with increasing dry matter intake than Hereford cows [63]. It seems that Hereford cows could not utilize the additional dry matter intake as efficiently for reproduction, lactation, or tissue growth, resulting in increased heat production.

Cow efficiency is a complex trait involving genetic potential for lactation, reproduction, and growth or mature size, as well as the ability of the cow to optimize nutrient partitioning to maintain herself, nurse a growing calf, and conceive the subsequent calf. Omics research is demonstrating that each of these biological functions requires a complex set of gene expression patterns and metabolic pathways, creating an omics signature for a genetic trait [21,22,23,24,25]. While mature size, growth potential, and milk production have been the focus of much cow efficiency research, the current analysis indicates that these traits do not adequately describe the complex biological functions that allow breeding females to adapt to their nutritional environment.

This analysis revealed several limitations of the BCSM and the underlying scientific literature. Reproductive efficiency appears to be a key driver of cow efficiency at lesser dry matter intake, and the inability of the BCSM to estimate the decline in reproductive efficiency is a major limitation. Mathematical modeling of reproduction is difficult, especially in the context of nutrition. The BCSM estimates postpartum interval based on body condition score using previous research as a guide [13]. However, the BCSM assumes the same probability of conceiving in each estrus cycle following the postpartum interval regardless of body condition score. Additionally, the BCSM assumes the same probability of pregnancy loss regardless of body condition score. Further research should evaluate the effect of energy status on follicular recruitment and ovulation and embryo loss with which to improve the BCSM.

Net energy for maintenance is computed in the BCSM based on body weight according to NASEM [32]. However, body composition affects maintenance energy requirements. Estimates of metabolizable energy required to maintain 1 kg of protein range from 95 to 193 kcal, and to maintain 1 kg of fat range from −7 to 51 kcal [91,92,101,102], indicating that cows with greater body fat relative to protein would have lesser maintenance energy requirements [60,62]. Additionally, visceral organs significantly contribute to maintenance energy requirements [72], and the level of feeding influences visceral organ mass [65,66] and heat production [59]. Freetly et al. [67] estimated changes in heat production with changes in feeding level, and diet characteristics are known to influence visceral organ mass [68,69,70,71], but there is a lack of comprehensive equations to predict maintenance energy requirements based on diet characteristics and feed intake. Van Milgen et al. [55] develop an equation to estimate fasting heat production in swine, separating muscle, viscera, and fat, which would allow adjustments for changing body composition, but a similar equation is not available for cattle. Additionally, there is a lack of equations necessary to estimate changes in visceral organ mass based on diet characteristics and feed intake.

The BCSM and other models [32,46] assume that cows will mobilize body tissue reserves to meet energy requirements for genetic maximum milk production, resulting in concomitant loss of body condition. However, energy intake has been shown to influence milk production and concentration of milk components [33,51,52,53,54]. Cows likely balance between maintaining body condition and milk production, as losing too much body condition would be detrimental to fitness and survivability. However, research and estimating equations to calculate the balance between body tissue loss and milk production with changing energy intake are lacking in the literature. Future modeling efforts should develop equations to estimate changes in milk yield and body tissue based on differences in energy intake.

Cow efficiency involves traits of mature size, growth potential, reproduction, and lactation, all of which are polygenic traits affected by many metabolic pathways [21,22,23,24,25]. Future modeling efforts could be useful to identify the combination of phenotypic characteristics important for improved cow efficiency and adaptation to the production environment. However, better compartmentalization and mathematical understanding of the biological functions of beef cows are necessary. For example, maintenance energy requirements are more complex than simply size or weight, involving body fat, protein, and visceral organ composition at the least. Modeling of these components independently, followed by aggregation of the individual energy requirements, will be necessary. Additionally, integration of genetic information such as genomic breeding values [103] and new computational approaches to animal breeding such as machine learning [104,105,106,107] could enhance biological system modeling through better representation of genetic variation to identify genotypes adapted to specific production environments.

## 5. Conclusions

The BCSM was not able to replicate the genotype–nutritional environment interaction on cow efficiency reported by Jenkins and Ferrell [20]. The grams of calf weaned per kg of dry matter intake per cow exposed followed a similar pattern with increasing dry matter intake regardless of genetic potential for milk production and growth, contrary to the results of Jenkins and Ferrell [20]. The nutrition component of the BCSM, built on NASEM [32] equations, did not adequately replicate the decrease in calving percentage associated with decreasing dry matter intake of cows with high genetic potential for milk yield and growth. Based on the work of Jenkins and Ferrell [20,33,62,63,90,99,100], breeds (i.e., genotypes) differ in the mechanisms by which they respond to increasing dry matter intake (i.e., nutritional environment), involving more than just genetic potential for milk production and mature size/growth. Future research should focus on understanding the variability in biological mechanisms by which genotypes respond to changing nutritional environments, leading to enhanced modeling of the cow-calf production system.

## Figures and Tables

**Table 1:** Reproduction parameters for 3+-year-old cow genotypes averaged across dry matter intake level.

MSBW (kg)	Peak Milk (kg/d)	Pregnancy (%)	Calving (%)	Weaning (%)
450	8	96.0	88.3	84.2
	10	95.7	88.2	84.2
	12	95.4	88.1	84.1
550	8	96.0	88.3	84.3
	10	95.8	88.3	84.2
	12	95.6	88.2	84.2
650	8	96.1	88.3	84.3
	10	95.9	88.3	84.3
	12	95.7	88.2	84.2

**Table 2:** Reproduction parameters for 3+-year-old cows at each of 4 dry matter intake levels averaged across genotypes.

Dry Matter Intake (g/kg^0.75^)	Pregnancy (%)	Calving (%)	Weaning (%)
58	85.5	84.2	80.4
76	95.0	88.1	84.1
93	96.2	88.4	84.3
111	96.2	88.4	84.3

**Table 3:** Progeny production parameters for 3+-year-old cow genotypes not impacted by dry matter intake level.

MSBW (kg)	Peak Milk (kg/d)	Birth Weight (kg)	Creep DMI (kg/d)	Weaning Age (d)	Pre-Weaning ADG (kg/d)	Weaning Weight (kg)	Weaning Weight per Cow Exposed (kg)
450	8	33.5	0.43	187	0.64	151	149
	10	33.5	0.45	186	0.76	173	170
	12	33.5	0.47	186	0.88	195	192
550	8	34.0	0.46	187	0.68	158	155
	10	34.0	0.48	187	0.81	181	178
	12	34.0	0.51	186	0.93	205	201
650	8	34.4	0.49	187	0.71	164	161
	10	34.4	0.52	187	0.84	188	185
	12	34.4	0.54	187	0.98	213	210

## Data Availability

Beef Cattle Systems Model output data are available upon request.

## Supplementary Materials

The following supporting information can be downloaded at https://www.mdpi.com/article/10.3390/ani16030372/s1, Table S1: Ingredient and nutrient composition of the diets used for each class of cattle in the simulations of the Beef Cattle Systems Model; Table S2: Empty body fat percentage, energy content of empty body weight change, and percentage of fat per kilogram of empty body weight change at each cow body condition score used in the Beef Cattle Systems Model; Equation (S1): Calculation of the net energy requirement for gestation of cows in the Beef Cattle Systems Model; Equation (S2): Calculation of the net energy requirement for lactation of cows in the Beef Cattle Systems Model; Equation (S3): Calculation of the net energy requirement for maintenance of cows in the Beef Cattle Systems Model.
