# Supplementary material for: Evaluation of the Beef Cattle Systems Model to Replicate a Beef Cow Genotype × Nutritional Environment Interaction
Source: Animals (Basel). 2026 Jan 24;16(3):372. doi: 10.3390/ani16030372 (PMC12897432; doi:10.3390/ani16030372)
Supplement: Supplementary file 1 [file animals-16-00372-s001.zip › animals-4067744-supplementary.pdf]

Supplementary Materials

Table S1. Ingredient and nutrient composition of diets used for each class of cattle in simulations of the Beef Cattle Systems Model

**Ingredient, DM%[1]**	**Nursing Calves**	**Replacement Heifers**	**Bred Heifers**	**2-yr-old Cows**	**3+-yr-old Cows**
Ground alfalfa hay	--	30.0	10.0	10.0	77.5
Bromegrass hay	10.0	45.0	--	90.0	--
Bluestem hay	--	--	90.0	--	--
Corn silage	--	--	--	--	5.0
Corn	--	--	--	--	17.5
Soybean hulls	--	25.0	--	--	--
Oats	90.0	--	--	--	--
**Nutrient, DM**					
Crude protein, %	13.07	12.30	7.22	9.29	16.06
NDF, %	32.70	58.55	67.20	63.60	36.40
ADF, %	16.60	39.40	42.00	39.30	27.44
Fat, %	4.91	1.75	1.32	1.59	1.99
Starch, %	40.76	2.32	2.55	2.64	16.55
ME, Mcal/kg	2.69	2.02	1.83	1.90	2.25
NEm, Mcal/kg	1.77	1.16	0.99	1.05	1.35
NEg, Mcal/kg	1.15	0.61	0.44	0.51	0.77

[1]DM = dry matter; NDF = neutral detergent fiber, ADF = acid detergent fiber, ME = metabolizable energy; NEm = net energy for maintenance, NEg = net energy for gain

Table S2. Empty body fat percentage, energy content of empty body weight change, and percentage of fat per kilogram of empty body weight change at each cow body condition score used in the Beef Cattle Systems Model

**Cow BCS[1]**	**EBF (%)**	**Energy (Mcal/kg change)**	**EBF (%/kg change)**
1	3.77	3.69	39.3
2	7.54	4.22	44.9
3	11.30	4.76	50.7
4	15.07	5.30	56.4
5	18.84	5.84	62.2
6	22.61	6.38	67.9
7	26.38	6.91	73.6
8	30.15	7.45	79.3
9	33.91	7.99	85.1

[1]BCS = body condition score; EBF = empty body fat

Equation S1. Calculation of the net energy requirement for gestation of cows in the Beef Cattle Systems Model.

$$NEy,\ Mcal/d = [CBW \times (0.05855 - 0.0000996 \times t) \times \exp(0.03233 \times t - 0.0000275 \times t^2)] \div 1000$$

$$MEy,\ Mcal/d = NEy \div 0.13 \qquad (S1)$$

$$NEm\_gest,\ Mcal/d = MEy \times km$$

where, NEy is the net energy for conceptus growth, CBW is calf birth weight in kg, t is day of gestation, MEy is metabolizable energy for conceptus growth, NEm_gest is net energy for gestation, and km is the efficiency of metabolizable energy use for maintenance computed as the ratio of diet net energy for maintenance concentration to diet metabolizable energy concentration.

Equation S2. Calculation of the net energy requirement for lactation of cows in the Beef Cattle Systems Model.

$$NEl,\ Mcal/d = (0.7285 + 0.0007 \times t - 0.00012 \times Milk \times t) \times Milk \qquad (S2)$$

where, NEl is net energy for lactation, t is day of lactation, and Milk is the milk production on day t in kg.

Equation S3. Calculation of the net energy requirement for maintenance of cows in the Beef Cattle Systems Model.

If lactating,

$$NEm,\ Mcal/d = \text{if } [PML \leq 7,\ ((0.0007 \times (20 - T) + (0.077 \times 1.2)) \div 1.12) \times CSBW^{.75} + NEm\_gest + NEl,$$
$$\text{else } (0.0007 \times (20 - T) + (0.077 \times 1.2)) \times CSBW^{.75} + NEm\_gest + NEl]$$

otherwise, (S2)

$$NEm,\ Mcal/d = \text{if } [PML \leq 7,\ ((0.0007 \times (20 - T) + 0.077) \div 1.12) \times CSBW^{.75} + NEm\_gest + NEl,$$
$$\text{else } (0.0007 \times (20 - T) + 0.077) \times CSBW^{.75} + NEm\_gest + NEl]$$

where, NEm is net energy for maintenance, PML is peak milk yield in kg/d, T is average daily temperature in °C, CSBW is cow shrunk body weight in kg, NEm_gest is net energy for gestation in Mcal from Eq. S1, and NEl is net energy for lactation in Mcal from Eq. S2.
